# Framework for identification and measurement of spillover effects in policy implementation: intended non-intended targeted non-targeted spillovers (INTENTS)

**DOI:** 10.1186/s43058-022-00280-8

**Published:** 2022-03-14

**Authors:** Igor Francetic, Rachel Meacock, Jack Elliott, Søren R. Kristensen, Phillip Britteon, David G. Lugo-Palacios, Paul Wilson, Matt Sutton

**Affiliations:** 1 Health Organization, Policy and Economics (HOPE) Research Group, Centre for Primary Care and Health Services Research, School of Health Sciences, University of Manchester, Manchester, UK; 2 Danish Centre for Health Economics, University of Southern Denmark, Odense, Denmark; 3 Centre for Health Policy, Institute of Global Health Innovation, Imperial College London, London, UK; 4 Department of Health Services Research and Policy, London School of Hygiene and Tropical Medicine, London, UK; 5 Centre for Primary Care and Health Services Research, School of Health Sciences, University of Manchester, Manchester, UK; 6 Melbourne Institute: Applied Economic and Social Research, University of Melbourne, Melbourne, Australia

**Keywords:** Health policy, Spillover effects, Evaluation, Healthcare economics and organisations, Programme evaluation, Unintended effects

## Abstract

**Background:**

There is increasing awareness among researchers and policymakers of the potential for healthcare interventions to have consequences beyond those initially intended. These unintended consequences or “spillover effects” result from the complex features of healthcare organisation and delivery and can either increase or decrease overall effectiveness. Their potential influence has important consequences for the design and evaluation of implementation strategies and for decision-making. However, consideration of spillovers remains partial and unsystematic. We develop a comprehensive framework for the identification and measurement of spillover effects resulting from changes to the way in which healthcare services are organised and delivered.

**Methods:**

We conducted a scoping review to map the existing literature on spillover effects in health and healthcare interventions and used the findings of this review to develop a comprehensive framework to identify and measure spillover effects.

**Results:**

The scoping review identified a wide range of different spillover effects, either experienced by agents not intentionally targeted by an intervention or representing unintended effects for targeted agents. Our scoping review revealed that spillover effects tend to be discussed in papers only when they are found to be statistically significant or might account for unexpected findings, rather than as a pre-specified feature of evaluation studies. This hinders the ability to assess all potential implications of a given policy or intervention. We propose a taxonomy of spillover effects, classified based on the outcome and the unit experiencing the effect: within-unit, between-unit, and diagonal spillover effects. We then present the INTENTS framework: Intended Non-intended TargEted Non-Targeted Spillovers. The INTENTS framework considers the units and outcomes which may be affected by an intervention and the mechanisms by which spillover effects are generated.

**Conclusions:**

The INTENTS framework provides a structured guide for researchers and policymakers when considering the potential effects that implementation strategies may generate, and the steps to take when designing and evaluating such interventions. Application of the INTENTS framework will enable spillover effects to be addressed appropriately in future evaluations and decision-making, ensuring that the full range of costs and benefits of interventions are correctly identified.

**Supplementary Information:**

The online version contains supplementary material available at 10.1186/s43058-022-00280-8.

Contributions to the literature

- Implementation strategies often have unintended consequences or “spillover” effects beyond those originally intended, yet little guidance exists on how to evaluate these.
- We provide conceptual clarity around the term “spillover” through a scoping review.
- A taxonomy of spillover effects is developed, classified based on the outcome and unit experiencing the effect.
- We present a comprehensive framework for the identification and measurement of spillover effects resulting from changes to the way healthcare services are organised and delivered.
- The INTENTS framework guides users through the potential effects that implementation strategies may generate and steps to take when designing and evaluating such interventions.

## Introduction

Among researchers and policymakers, there is increasing awareness of the potential for the implementation of health interventions and policies to have consequences beyond those initially intended. For example, the Medical Research Council guidance on complex interventions emphasises that these unintended consequences should be considered where possible but provides little guidance on *how* best to do this systematically [1]. This lack of direction is also evident in the implementation science literature. For example, the recent ImpRes Framework encourages research teams to be “mindful” and to explore the potential unintended consequences of implementation efforts but does not say how [2]. There is also recognition that successful de-implementation at one level may also lead to potentially negative unintended consequences at another level, but how best to mitigate or prevent occurrence is not yet well understood [3].

A growing body of literature now uses the terms “spillover effects” or “spillovers” for such wider impacts [4]. This term is particularly prevalent in the areas of psychology, economics, and programme evaluation, where efforts are often made to quantify spillover effects resulting from interventions. This term is neutral in its connotation, focusing equally on desirable and undesirable consequences not intended at the outset. An obvious spillover occurs because the implementation of interventions and policies requires redeployment of existing resources or use of new resources that could otherwise have been used elsewhere [5]. Other spillovers may arise because of learning or because inputs are involved in multiple activities. The growing literature on spillover effects could provide useful lessons on how to identify and capture these broader effects, informing the development of practical recommendations missing from current guidance.

The identification and measurement of spillovers remain partial and unsystematic. Spillover effects have been examined in relation to pay-for-performance schemes [6, 7] and target waiting times [8, 9] in England. More recently, in relation to the 2010 Affordable Care Act in the USA, studies reported on spillover effects resulting from the expansion of insurance coverage [10, 11] and alternative provider payment models [12, 13].

We develop a framework for the identification and measurement of spillover effects resulting from policy implementation. This framework provides a structured approach to guide consideration of spillover effects and to enable optimal intervention design, maximising the desired outcomes whilst minimising the potential for negative unintended consequences. It is intended for use both when designing interventions and when planning evaluations of their impacts.

## Methods

To inform the development of the framework, we conducted a scoping review to map the existing literature on spillover effects in health and healthcare interventions generally.

### Scoping review of the literature

We sought to identify and synthesise evidence from studies on both healthcare and broader interventions targeting health and health behaviours (public health, non-pharmaceutical or non-healthcare interventions, health education, etc.). In devising and conducting the search, screening, and synthesis procedures, we adopted the original and updated methodological frameworks for scoping reviews by Arksey and O’Malley [14] and Levac et al. [15]. Scoping reviews offer an appropriate methodology for mapping the field in terms of the existing evidence [15]. We chose a scoping review rather than a systematic literature review because we aimed to gain a conceptual understanding of spillovers [16]. We reported the study according to the new guidelines for scoping reviews [17]. The checklist is available in Additional file 1.

We searched the online databases EMBASE and MEDLINE from January 1991 to September 2020. We focused on these two databases with the goal to strike a reasonable balance between breadth of coverage of the health and healthcare literature and the number of papers to scope for a non-systematic review. We judged this to be sufficiently wide to capture the evolution of the use of the term “spillover” over time and across different journals and branches of the literature. The start date of January 1991 was chosen in order to have a consistent version of the *Journal of Economic Literature* (JEL) classification system codes available for filtering purposes, as these were updated and expanded after 1990. We excluded papers addressing biological, physiological, and zoonotic spillovers, as the term spillovers is used to refer to a very different and unrelated concept in these literatures. Only full articles in English were included. The full search strategy is available in Additional file 2. After removing duplicates, we conducted the first screening of titles and abstracts for relevance to our inclusion criteria. The second stage involved full-text screening of the remaining articles. At both stages, two reviewers screened the articles independently and resolved any inconsistencies bilaterally.

We focused on articles discussing—primarily or secondarily—at least one spillover effect associated with outcomes of a specific health or healthcare intervention. Therefore, we excluded studies related to spillover effects in other domains (e.g. biology, trade). In the same spirit, we also excluded all editorials, letter to editors, dissertations and review articles, and favouring empirical studies which offered more detail into the analytical approach towards spillovers. We defined further detailed inclusion criteria for our scoping review iteratively. Our initial exploration revealed that a number of papers were using the term spillovers in passing, but did not actually examine the spillover effects in any detail. For example, the possible existence of spillover effects was mentioned only as a potential explanation for unexpected effects measured in the analysis, or the potential for spillover effects was discussed broadly without referring to specific cases. Therefore, we excluded all such articles which mentioned the term “spillover” incidentally or without actually discussing it. Furthermore, we excluded association studies and focused only on articles analysing interventions of some kind. Since our interest lay precisely in how spillover effects have been analysed, we selected intervention studies irrespective of their analytical design (including trials, quasi-experimental, and observational studies).

The selected articles were fully reviewed and details extracted regarding the following set of salient characteristics of the spillover effects: type of intervention generating spillover effects, units involved, whether the spillover effect was measured, whether and how the mechanisms driving the spillover effect were discussed, and the evaluation design used.

### Framework development and validation

Based on the evidence synthesised by the scoping review and building on a limited base of literature on the categorisation of spillover effects [18–21] from different subjects or disciplinary areas, we developed a framework for the identification and measurement of spillover effects. The framework was developed, refined, and validated collectively by all co-authors. During the first joint session, we discussed the validity and relevance of all steps in a draft framework proposed by the lead author and assessed concordance in the classifications of spillover effects based on samples of articles randomly assigned to each co-author. Building on the feedback from the first round, we refined the framework and repeated the exercise, framing the discussion around the categorisation of a new random sample of papers assigned to each co-author participating in the exercise.

## Results

### Scoping review summary

**Fig. 1:**
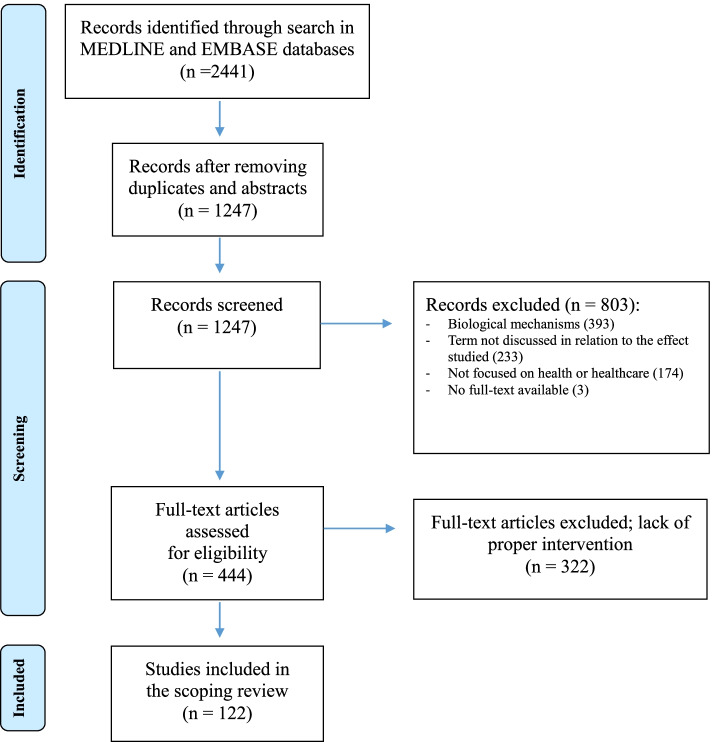
Literature search and selection stages

Figure 1 shows the flow diagram describing all stages of article search and selection. The initial search produced 2441 results. After removing duplicates and conference abstracts, we screened the titles and abstracts for 1247 unique articles. Overall, 1125 articles were excluded, resulting in the inclusion of 122 articles (see Additional file 3 for the full list of selected papers).

The number of papers matching our inclusion criteria is considerably higher for papers published in the last decade. This finding is consistent with a growth in the volume of published literature addressing spillover effects, although publication volumes in general have also increased during this period.

The selected studies discuss a wide range of interventions in health and healthcare, of various types (including training, regulation, financial incentives, direct provision of healthcare) and affecting subjects at various levels which may not be the same as those targeted by the main intervention (including patients, providers, healthcare organisations). Table 1 groups the interventions featured in the selected literature according to the Cochrane Effective Practice and Organisation of Care (EPOC) taxonomy [22, 23]. Besides synthesising an otherwise wide range of interventions, the grouping is useful to detect potential recurrences between spillover effects generated by different types of interventions.

**Table 1:** Type of interventions featured in the articles included in the scoping review, grouped according to the EPOC taxonomy

EPOC taxonomy	Number of papers	Types of interventions featured in articles
**Financial arrangements** Changes in how funds are collected, insurance schemes, how services are purchased, and the use of targeted financial incentives or disincentives	57	Hospital readmission reduction programme, results-based financing, provider payment models or fees, disease-specific financial incentive programmes, financial interventions, insurance coverage expansion, changes in healthcare prices or coverage for patients
**Implementation strategies** Interventions designed to bring about changes in healthcare organisations, the behaviour of healthcare professionals, or the use of health services by healthcare recipients^a^	27	Capability-enhancing interventions (training, health promotion, health communication)
**Governance arrangements** Rules or processes that affect the way in which powers are exercised, particularly with regard to authority, accountability, openness, participation, and coherence	21	Prescribing recommendations/guidelines or changes to prescription drugs lists, extended access to treatment, gatekeeping/managed care, treatment guidelines, public reporting of hospital output/quality information, extended healthcare benefits for specific populations
**Delivery arrangements** Changes in how, when, and where healthcare is organised and delivered and who delivers healthcare.	17	Closure/opening of hospitals/wards or drug stores, e-health technology, provider/caregiver training, changes in the care process, screening programmes, public health measures (bednets, purified water, living conditions, etc.), clinical and similar interventions

^a^We have been generous in our interpretation of studies in line with the EPOC taxonomy but recognise that many of these are not clearly defined strategies

Most selected articles (98 of 122) report a statistically significant spillover effect. Consistent with a generalised bias towards the publication of studies reporting significant effects, this can be interpreted as a broad signal that spillover effects are discussed in papers only when they are found to be significant rather than as a pre-specified feature of evaluation studies. This does not favour a comprehensive evaluation of all implications of policy interventions. If only the studies featuring a statistically significant spillover effect are reported in the literature, policymakers and researchers may make decisions based on a partial overview of the evidence. Null results can still be informative, for example, providing reassurance that interventions do not appear to generate negative spillover effects. Potential impacts may also be missed if studies are underpowered to detect significant effects.

A second important observation emerging from our review is that the spillover effects reported in the identified literature are either experienced by agents or units not originally targeted by the intervention, or concern impacts on outcomes for the targeted agents/units not originally envisaged. This suggests an important distinction about the nature of spillover effects based on the units involved and the extent to which the spillover effect is similar to or different from the intended goal of the intervention. This distinction is summarised in the simple matrix in Table 2.

**Table 2:** A taxonomy of spillover effects in relation to the main intended intervention effect

		Type of effect	
		Intended (by the intervention)	Non-intended (by the intervention)
**Unit affected**	Targeted by the intervention	Main effect	*Within-unit* Spillover effect
	Not targeted by the intervention	*Between-units* Spillover effect	*Diagonal* Spillover effect

On the one hand, interventions can also generate non-intended effects on the units targeted by the intervention, i.e. impacts on outcomes other than those intended by design, which we term within-unit spillovers. For example, a scheme incentivising providers to improve diagnosis of dementia was found to incidentally reduce the time providers allocated to activities related to patients admitted for other health conditions, but also to improve providers’ efforts to raise quality in other care areas [24]. Likewise, some studies found that the Hospital Readmission Reduction Program yielded positive results in terms of readmissions beyond the procedures specifically penalised, therefore improving outcomes for non-targeted patients [25, 26]. Additionally, direct consumer advertising of medicines had the unintended consequence of triggering learning effects and increased willingness to interact with physicians [27]. On this point, it is worth specifying that we define spillovers from the perspective of the unit targeted by the intervention, rather than the unit experiencing the end outcome. For example, if a healthcare organisation is targeted, a change in provider behaviour can lead to a within-unit spillover onto patient outcomes.

On the other hand, the intended effect of a given intervention can affect units that were not initially targeted [21], which we term between-units spillovers in Table 2. Examples of interventions featuring such spillover effects include a cluster-randomised trial promoting the use of insecticide-treated bednets where the protective effects of bednets extended beyond the households using them [28], asset or conditional cash transfer programmes where non-treated households benefitted from the transfers either through friendship/kinship networks or through other market-mediated channels [29–31], a body mass reduction programme for overweight individuals [32] and a colorectal cancer screening programme spilling over to non-treated individuals through social networks [33].

Furthermore, non-targeted units can also experience effects of interventions that are different from the intended goals of the programme originating them, which we call diagonal spillovers. At first sight, diagonal spillovers may appear similar to between-units spillovers in that the intervention effect extends to non-targeted units. However, we distinguish them from between-units spillovers based on the outcome affected: diagonal spillovers refer to instances where an intervention on a targeted unit generates a non-intended effect on a non-treated unit. Examples of diagonal spillovers include changes in Medicare provider fees generating non-intended effects on patients covered by different health insurance schemes [34], changes in prescription drug lists reimbursed by Medicare part D affecting younger population through intensified advertising of newly included pharmaceutical products [35], or the introduction of mental health parity laws triggering increases in private insurance premiums and higher enrolment in public health insurance [36]. In the literature reviewed, diagonal spillover effects seemed more pronounced for mediated interventions, i.e. interventions that were implemented at one level of the health system with the intent of producing effects on units at a different level in the system. Examples of mediated interventions are a change in Medicare reimbursement fees affecting quality and quantity of healthcare provision for commercially insured patients, or the extension of the list of prescription drugs covered by Medicare part D causing an increase in pharmaceutical advertisement and consumption among the non-elderly patients (not covered by Medicare). Overall, classifying both the units that could be affected and the domains upon which they may be affected is a crucial first step in mapping out the potential spillover effects that may occur as a result of an intervention.

In light of the above and considering that any given intervention can be associated with multiple types of spillover effects, in Table 3, we map the literature reviewed according to the general taxonomy in Table 2. With the aim of providing a mapping between main and spillover effects observed in the literature, we also report the frequency of different intervention groups within each spillover class.

**Table 3:** Classes of spillovers and interventions examined in the reviewed literature

Classes of spillovers^**a**^	Number of papers^**a**^	Underlying intervention groups observed in the literature as introduced in Table **2**^**a)**^
*Within-unit* spillover effect Non-intended effect on targeted unit^b)^	55	.Financial arrangements (25) .Governance arrangements (12) .Implementation strategies (11) .Delivery arrangements (7)
*Diagonal* spillover effect Non-intended effect on non-targeted unit^b)^	38	.Financial arrangements (26) .Governance arrangements (6) .Implementation strategies (3) .Delivery arrangements (3)
*Between-units* spillover effect Intended effect on non-targeted unit^c)^	36	.Implementation strategies (15) .Financial arrangements (10) .Delivery arrangements (8) .Governance arrangements (3)

^a^ Seven articles featured spillover effects classified into two classes; hence, the 129 spillover effects classified in spite of 122 articles reviewed

^b^ Articles included in this class discussed spillovers in the form of effects different from those initially intended for the intervention, extending either on the same target unit (i.e. treatment) or to the non-targeted unit (i.e. control)

^c^ Articles included in this class discussed spillovers in the form of main intended effects of the intervention extending beyond the unit initially targeted (i.e. the treatment unit)

Overall, our scoping review suggests that some intervention types are more likely to lead to the reporting of certain types of spillovers in the published literature. For example, interventions related to financial arrangements were more likely to be reported to generate within-unit or diagonal spillovers. In contrast, studies examining policies addressing implementation strategies most often report between-units spillovers.

### Spillover mechanisms identified in the reviewed articles

Understanding the mechanisms which generate spillover effects is key if these are to be harnessed to encourage positive spillovers and mitigate negative ones. We identified an interesting recurrence in terms of the spillover mechanisms mentioned in the studies (Table 4). Among the studies addressing spillover effects occurring between units, about half suggested multiplying effects related to the spread of information through social networks. Other recurring mechanisms were spatial dependencies driven by closeness or common social and environmental factors, as well as effects driven by shared resources in healthcare activities involving providers treating multiple groups of patients.

**Table 4:** Recurrent mechanisms reported to explain the different classes of spillovers in the reviewed literature

Classes of spillovers	Number of papers	Direction of spillover^**a)**^	Type of mechanism
*Within-unit*	55	Favourable: 35 Unfavourable: 9 Both: 1 Null: 10	.Learning effects, improvement in overall skills/knowledge/awareness
			.Chain response in related behaviours (e.g. physical activity and food intake)
			.Complementarity or substitution (in use of inputs or consumption of goods/services) in response to changes in prices or quantities
			.Shared resources, complementary activities, shared fixed costs
			.Excessively bureaucratised or structured procedures generating changes in others
*Diagonal*	38	Favourable: 21 Unfavourable: 5 Both: 2 Null: 10	.Complementarity or substitution (in use of inputs or consumption of goods/services) in response to changes in prices or quantities
			.Spatial diffusion of the effect (due to ecological mechanisms, proximity, overlapping catchment areas, etc.)
			.Shared resources, complementary activities, shared fixed costs
			.Social referencing, social learning
			.Learning effects, improvement in overall skills/knowledge/awareness
			.“Welcome-mat” effect (entering a social protection scheme improves the likelihood of getting access to others through increased knowledge of rules, regulations, availability, etc. for same individuals and family members or friends)
*Between-units*	36	Favourable: 27 Unfavourable: 4 Both: 1 Null: 4	.Social network effect, social learning, “word of mouth”
			.Spatial diffusion of the effect (due to ecological mechanisms, proximity, overlapping catchment areas, etc.)
			.Shared resources, complementary activities, shared fixed costs
			.“Welcome-mat” effect (entering a social protection scheme improves the likelihood of getting access to others through increased knowledge of rules, regulations, availability, etc. for the same individuals and family members or friends)

^a^ The interpretation of the spillover direction is inferred from the papers’ explicit interpretation. We used the favourable/unfavourable spillover contraposition (instead of positive/negative) to avoid misunderstandings based on the effect sign

In contrast, the articles pointed to various different mechanisms to explain spillovers which we classified as within-unit or diagonal, with none standing out as the predominant explanation. Examples include unanticipated behavioural responses to treatment by both patients and providers, complementarities or substitution in healthcare production (e.g. bundled care), effort diversion, gaming, and complementarity or substitution effects. In Table 4, we also summarise the direction of the spillover effects, highlighting that in the reviewed literature most studies found favourable (or “positive” spillover effects).

Examining the mechanisms hypothesised to cause observed spillover effects is necessary in order to shed light on the nature of spillover effects and hence help in gauging their relevance for the evaluation of the underlying intervention. For example, in studies discussing health insurance coverage expansion, income spillover effects onto non-healthcare consumption patterns may or may not be policy-relevant, depending on the goals of the policy itself (e.g. financial health protection for the poor). On the other hand, income spillover effects associated with conditional cash transfer for health onto consumption of other goods or services may be less interesting for health policy evaluation purposes.

Understanding the mechanisms that have been found to underpin spillover effects in the past is also helpful both for designing interventions and subsequent evaluations going forward. Knowledge and evidence on mechanisms can inform the design of interventions to maximise positive spillover effects whilst minimising negative ones, and guide the groups and outcomes that should be examined in the subsequent evaluation in order to capture an interventions’ full effects using an unaffected comparator group.

This classification of spillover types, along with mechanisms identified as driving spillover effects to date, should serve as a useful basis for researchers wishing to map out the potential spillover effects that may occur as a result of an intervention.

### Developing a framework to classify spillover effects: the INTENTS framework

Building on the elements above, we designed a step-wise guide for the identification and measurement of spillovers resulting from health and healthcare interventions, which we named the Intended Non-intended TargEted Non-Targeted Spillovers (INTENTS) framework. This framework is designed to be a useful starting point for researchers, practitioners, and policymakers when selecting a sensible evaluation design for an intervention, prospectively. Potentially, a similar step-wise process may also be informative at the stage of intervention design, to explicitly map potential spillover effects and calibrate an intervention to maximise its benefits, alongside the development of theories of change or theory-based evaluation frameworks. Likewise, the INTENTS framework may be useful to update evaluation plans mid-way through the implementation of an intervention based on early empirical evidence or conceptual understanding. In the next paragraphs, we describe the INTENTS framework step by step, using examples to clarify the meaning of the general activities proposed at each step. In Table 5, we summarise the INTENTS framework, whilst the following sections provide additional details on its different elements.

**Table 5:** The INTENTS framework for classifying spillover effects

*Step #1: What are the expected outcomes of the intervention?* *Step #2: At what level can spillover effects take place?* a) *Who is targeted by the intervention?* b) *Who is expected to change behaviour as a result of the intervention?* c) *Whose behaviour/outcomes may change as a result of the intervention?* *Step #3: Which spillover effects could the intervention generate?* a) *Within-unit spillover effects (non-intended effect on a targeted unit)* b) *Between-units spillover effects (intended effect on a non-targeted unit)* c) *Diagonal spillover effects (non-intended effect on a non-targeted unit)* *Step #4: What is the nature of the potential spillover effects identified in Step #3?* a) *Is the spillover effect really different from the intended outcome?* b) *Is the spillover relevant and related to the goals of the intervention?* c) *Is the spillover effect consistent with the time frame of the intervention?* d) *Is there a credible mechanism for the spillover?*	

#### Some preliminary definitions

The use of the INTENTS framework presumes that the intervention in question is related to health or healthcare. The goal is to formulate hypotheses in relation to potential wider effects which may result from the intervention. This can help to determine prospectively whether the intervention is likely to generate spillover effects which should be fostered or mitigated, as well as to decide whether to consider these potential spillovers in subsequent evaluations of the intervention, both in terms of overall effectiveness and evaluation design.

Before describing the framework steps, we define a few key concepts used in the remainder of the paper, based on the taxonomy represented in Table 2.

##### Intended and non-intended effects

Throughout the description of the framework, we define “intended effects” as the outcomes which are expected to change by whoever designs an intervention, as a result of the implementation of the intervention itself. By extension, we define “non-intended effects” as all other outcomes that the intervention does not explicitly aim to change, but could nonetheless be affected. The type of outcomes classified as non-intended effects depends on the unit targeted by the intervention. For example, in the case of an intervention targeting a primary care practice in relation to a specific group of patients (e.g. patients with chronic conditions), one could classify as non-intended effects both the main performance criterion addressed by the intervention measured on a different (non-targeted) group of patients and different performance criteria measured on the same patients with chronic conditions.

##### Targeted and non-targeted units

Any intervention typically defines a “targeted” unit, i.e. a unit that the intervention explicitly aims to affect and which should experience a change in outcomes as a result. In healthcare, the treated units are typically individual or groups of patients, healthcare workers, healthcare organisations, and geographic areas. “Non-targeted” units then refer to units that the intervention is not explicitly aiming to affect and that should therefore not experience changes in outcomes associated with the implementation of the intervention.

#### Overview of framework steps

In this section, we discuss the components of the INTENTS framework, expanding on the questions to ask at each step and their rationale. The section is intended to be read alongside Table 7, which includes an illustrative application of the INTENTS framework to the case of the Quality and Outcomes Framework, a pay-for-performance scheme introduced to improve quality across general practices in the UK [37].

##### Step #1: what are the expected outcomes of the intervention?

The first crucial step entails understanding the aims of the intervention and the outcomes targeted for impact. This can be achieved through triangulation of different methods, for example:

- Intervention mapping with key informants [38–41]
- Review of related literature [42, 43]
- Review of relevant policy documents and high-level gap maps [44, 45]
- Theories of change and negative programme theory [46, 47]

Once a list of outcomes or a logic model resulting from a thorough effort in mapping potential intervention effects is available, one can start to iteratively apply steps 2 to 4 of the INTENTS framework.

##### Step #2: at what level can spillover effects take place?

Interventions can impact multiple units or actors beyond those explicitly targeted. Some interventions are implemented through units at a given level to affect units at a different level, upwards or downwards in the health system hierarchy (see Table 6 for some examples). Other interventions are implemented to directly affect the targeted units, without intermediary units or without affecting units at different levels. Hence, the characteristics of the intervention demarcate the type of spillover effect that can emerge.

**Table 6:** Examples of health system hierarchical levels to be considered in the framework

Health system level	Description
*Government authorities, regulators, administrative authorities, policymakers*	Central government, legislators, regions, counties, districts, local authorities, etc.
*Payers*	Health insurance, health funds, social security, government authorities
*Suppliers*	Pharma companies, manufacturers of medical devices and medical supplies, labs, etc.
*Healthcare organisations*	GP practice, healthcare organisation, ambulatory, hospital, pharmacy, nursing homes
*Individual healthcare providers*	Independent physiotherapists, independent dentists, GPs, clinicians, specialists, nurses, support staff, etc.
*Individuals/citizens/patients*	Including individual patients and their relatives, friends, social networks, other patients and their families

Source: Gilson [48], Savedoff and Hussman [49]

We first consider an intervention addressing the targeted unit directly, without intermediary units at different health system levels. This intervention design ensures that no unit at other levels in the hierarchy is involved in the implementation of the intervention and hence changes behaviour as a result of the intervention. Assuming that the targeted unit also represents the endpoint of the healthcare process addressed by the policy (i.e. there are no cascading relationships), this type of intervention is more likely to generate spillover effects only within the targeted units, or among other units at the same health system level as the targeted units, which have some kind of interaction with the latter. By the characteristics of the intervention, none of these potential spillover effects would be mediated by intermediary units, which is why we define these spillover effects as non-mediated.

On the other hand, interventions targeting a unit at a different hierarchical level (either upwards or downwards) than the level at which the intervention is implemented can additionally generate what we define as mediated spillovers, akin to the mediated pathway of the intervention itself. The units experiencing a mediated spillover effect are typically linked to the targeted units by a specific agency, market or other known transmission mechanisms implicit to the intervention. To properly unpick these details, alongside the step suggested by detailed behaviour specification frameworks (e.g. the AACTT framework [50]), the questions to ask at this stage are as follows:

- Who is targeted by the intervention?
- Who is expected to change behaviour as a result of the intervention?
- Whose behaviour or outcomes may change as a result of the intervention?

In the answers to the questions above, if only one health system level is involved, the intervention can typically only generate non-mediated spillover effects. Instead, if two or more health systems levels are involved, mediated and non-mediated spillover effects may result from implementation. The distinction can be useful to discriminate mechanisms and to better understand the intervention itself and the possible trajectories in terms of spillover effects.

##### Step #3: which spillover effects could the intervention generate?

In conceiving any healthcare intervention, designers should do their best in considering all possible spillover effects that may be generated. This can be achieved through a comprehensive mapping of all processes and pathways associated to the changes brought by the intervention, as suggested by step #1. Building on the preliminary definitions above and in Tables 2 and 3, we can classify spillover effects generated by healthcare interventions into three main classes:

- Within-unit spillover effects (non-intended effect on a targeted unit)
- Between-units spillover effects (intended effect on a non-targeted unit)
- Diagonal spillover effects (non-intended effect on a non-targeted unit)

The choice is not limited to one—an intervention may generate multiple types of spillover effects. Table 3 provides a useful overview of the type of interventions found to be associated with different classes of spillover effects in the literature to date. Furthermore, step #2 helps in defining the units that could potentially experience spillover effects and thus demarcates the potential search for spillovers. The goal of this step is to obtain a list of potential spillover effects that may result from the intervention as designed. These spillover effects may be positive, and hence worth encouraging, or negative and thus to be discouraged. In listing the potential spillovers emerging from step #3, it is helpful to formulate hypotheses on the direction of the effects. This can be either informed by step #1 or previous literature.

##### Step #4: what is the nature of the potential spillover effects identified in step #3?

Given a list of spillover effects defined in step #3, the elements proposed below help in distinguishing potential spillover effects that are relevant from a policy evaluation perspective and reflecting on their nature. The questions to ask are as follows:

- *Is the spillover effect really different from the intended outcome?* For the sake of a comprehensive but coherent evaluation, the spillover effects considered should be sufficiently dissimilar from the intended outcome of the intervention, so that it constitutes a separate but associated effect. To this end, the spillover effect should not be conceptually equivalent nor sequentially related to the target outcome, for example, via a direct clinical pathway or because a process is targeted to affect an outcome downstream.
- *Is the spillover relevant and related to the goals of the intervention?* Spillover effects, whilst conceptually distinct from the intended effect, should share the same underlying motives, drivers and scope as the intended effect (see Galizzi and Withmarsh [4] for a useful discussion of this argument). Outcomes that do not share the same motives as the intended effect—either randomly or broadly triggered by the intervention of interest—are likely to reveal associations that are not relevant for an analysis of spillovers in health settings.
- *Is the spillover effect consistent with the time frame of the intervention?* Timing in the effect also helps to disentangle spillover effects from long-run consequences or to identify inconsistencies with the timing of the intervention. In the spirit of programme evaluation, the existence of spillover effects should be evaluated over a time horizon consistent with the intervention. Effects extending far beyond this time horizon may be medium to long-run effects of the intervention. Therefore, it is worth taking into account that mediated spillover effects experienced upwards in the hierarchy (e.g. change in guidelines in few local authorities triggering changes in regulation, patient-level behavioural interventions changing workflows for primary care practitioners) are more likely to develop over longer time periods. The specific time frame of the intervention is not always easy to establish when it is not explicitly defined, for example in the case of new policies or regulations introduced permanently (e.g. the Quality and Outcomes Framework in the UK or different components of the Affordable Care Act), and therefore, some discretion based on experience and case-specific knowledge is needed.
- *Is there a credible mechanism for the spillover?* Reflecting on the mechanisms driving potential spillovers may aid understanding of the impact pathways of the intervention, potentially improving the design of the intervention itself, for example, knowingly allowing the intervention to extend beyond the target group when this is desirable. The examples listed in Table 4 may offer a useful overview of some potential spillover mechanisms, which may need to be complemented with adequate theoretical concepts depending on the specific intervention area.

### A guided example of application of the INTENTS framework

Working through the INTENTS framework should result in a thorough understanding of the potential spillover effects which may be generated by a given healthcare intervention. This includes the units and outcomes which may be affected, and the mechanisms by which spillover effects could be generated. This structured approach should then form the basis for analysis planning, and potential intervention re-design.

In Table 7, we present an illustrative example of the application of these steps, using the UK Quality and Outcomes Framework (QOF), the longest running national primary care pay-for-performance scheme in the world [7, 51] that was first introduced across the UK in 2004. General practices report their performance on a large number of clinical indicators and receive financial bonus payments based on this performance. The clinical indicators refer to patients with specific diagnosis codes, clearly defining the targeted patients whose care contributes to a practice’s performance. A substantial literature has shown that care processes improved for these patients on the incentivised aspects of care, suggesting that the policy achieved the targeted effect [37, 52, 53]. However, the exploration of its unintended consequences or spillover effects has not been approached comprehensively or systematically.

**Table 7:** Application of the INTENTS framework to the case of the Quality and Outcomes Framework (QOF) in the UK

Framework step	Questions to ask	Comments in relation to the QOF
1	What are the expected outcomes of the intervention?	Increased primary care professional effort and therefore better performance on specified indicators of quality of care for patients with chronic conditions, resulting in improved clinical outcomes for these targeted patients.
2	At what level can spillover effects take place?	
	a) Who is targeted by the intervention?	All UK primary care professionals.
	b) Who is expected to change behaviour as a result of the intervention?	All UK primary care professionals.
	c) Whose behaviour/outcomes may change as a result of the intervention?	All UK primary care professionals and their registered patients.
3	Which spillover effects could the intervention generate?	
	a) Within-unit spillover effects (non-intended effect on a targeted unit)	Changes to primary care professional effort and performance on not-specified aspects of quality of care for targeted patients. For example, the QOF directly incentivised the recording of certain risk factors (including smoking status) for targeted patients. Targeted patients were found to have experienced positive spillover effects as primary care professionals also increased their recording of other clinically effective risk factors (BMI and alcohol consumption) for which they were not financially rewarded for these patients [7].
	b) Between-units spillover effects (intended effect on a non-targeted unit)	Changes to primary care professional effort and performance on specified indicators of quality of care for non-targeted patients. For example, the QOF directly incentivised the recording of certain risk factors (including smoking status) for targeted patients. Untargeted patients (those without the specific diagnosis codes targeted) were found to have experienced positive spillover effects as general practitioners also increased their recording of specified risk factors for patients not targeted by the policy [7, 54].
	c) Diagonal spillover effects (non-intended effect on a non-targeted unit)	Changes to primary care professional effort and performance on not-specified aspects of quality of care for non-targeted patients. For example, diagonal spillovers could have occurred if untargeted patients (those without the stated diagnosis codes) were found to have experienced changes in not-specified aspects of care quality (such as the recording of clinically effective but unincentivised risk factors, including BMI and alcohol consumption).
4	What is the nature of the potential spillover effects identified in step #3?	
	a) Is the spillover effect really different from the intended outcome?	Yes. The QOF considers specific indicators of care for targeted patient groups. Any changes in primary care professional effort and performance either on these indicators but for non-targeted patients, or on other aspects of care quality for targeted patients, represent separate effects to those intended by the policy.
	b) Is the spillover relevant and related to the goals of the intervention?	Yes. Wider changes in the quality of primary care services provided to patients are relevant and related to the goals of the QOF [51, 55].
	c) Is the spillover effect consistent with the time frame of the intervention?	Yes. The spillover effects were examined and detected over the same period as the direct effects of the policy on the recording of incentivised clinical indicators for targeted patients. Trends in recruitment and retention in primary care could, at the margin, be influenced by the effects of the Quality and Outcomes Framework on the attractiveness of working in primary care, but this would be difficult to isolate from more proximal influences [56–59].
	d) Is there a credible mechanism for the spillover?	The detected positive spillover effects from the QOF are consistent with the policy inducing general practices to make investments in quality that extended beyond the scheme. Complementarity in the production of healthcare appears to be a credible mechanism in this instance. The potential for negative spillover effects due to multi-tasking concerns around effort diversion (away from untargeted patients and aspects of quality of care not specified by the incentive scheme) was hypothesised. Whilst a credible mechanism, the evidence to date does not suggest that this effect dominated in practice [60]

## Discussion

### Summary of findings

There is an increasing awareness among both researchers and policymakers of the potential for the implementation of healthcare interventions to have consequences beyond those initially intended. Despite guidance emphasising the importance of evaluating such wider effects, recommendations for how to do so are lacking. We reviewed the literature on spillover effects in order to better understand the use of the term and the ways in which researchers have attempted to identify and measure such effects in practice. We found that spillover effects are frequently cited as explanations for unexpected research findings, but the current approach to their identification and measurement is sporadic and unsystematic.

Depending on their nature, spillover effects can either increase or decrease the overall effectiveness of interventions. The potential existence of spillover effects therefore has important consequences for the design and evaluation of implementation interventions and for decision-making. This is perhaps best highlighted by recent calls for strategies to mitigate adverse impacts of well-intentioned interventions for COVID-19, such as inequalities in access to healthcare or inequitable uptake or treatment for vulnerable/ marginalised populations [61]. Early and systematic assessment of the potential implications of a given intervention could have provided decision makers with earlier warnings and opportunities for faster corrective action. To this end, the INTENTS framework outlined here provides a structured means for researchers and policymakers to think systematically about the potential spillover effects that interventions in healthcare may cause, and crucially the steps they may take to mitigate when attempting to design and evaluate interventions from the outset.

In terms of evaluation design, the potential for spillover effects means that it may be necessary to widen the scope of evaluations and look beyond the targeted outcomes or recipients in order to assess the full impact. Failure to do so could result in over- or under-estimation of the impact of interventions. Secondly, spillovers can invalidate a key assumption for identifying counterfactuals in comparative evaluations because patients, professionals, services, and organisations not explicitly targeted by the programme could nonetheless be affected and hence fail to represent valid “controls”.

An interesting ancillary result of our scoping review is the extent to which we found the term spillover effects to have been applied to describe effects unsuitable to the definition provided by our INTENTS framework. In presenting our results, we make the point that spillover effects are distinct from the main intended effects of the interventions generating them. We also argue that spillover effects need to be gauged in terms of their relevance to the objectives of the original policy.

To this end, we highlight the underlying mechanisms driving spillovers and the time frame over which they may appear as important discriminants. Using this definition, our scoping review of the literature suggests that 24 of the 122 studies could be said to improperly use the spillover label for the effects they studied. Broadly, these studies involved outcomes that we found to be indistinguishable from the main intended goals of the intervention (11 of 24 studies), income effects that can be seen as implicit to the policy evaluated (9 studies, for example, related to the effect of sponsored health insurance coverage), and outcomes that are too far from the objectives of the intervention to appear relevant for the evaluation of the policy itself (4 studies). The application of the INTENTS framework will enable consistency in the assessment of spillover effects going forward, facilitating the appropriate classification of intervention effects.

### Strengths and limitations

Our study is the first to present a systematic overview of the use of the term “spillovers” in the healthcare setting and to develop a framework to aid a more systematic consideration of spillover effects in future research and policymaking. With these elements, our contribution offers new insights and a practical framework, adding to a limited body of existing conceptual literature on spillover effects in health and healthcare [18–21].

There are, however, limitations to our approach. Our study presents an overview of studies explicitly mentioning the term “spillover(s)”, rather than including all possible synonyms that may be used to convey a similar concept. It is exactly for this reason that we chose a scoping review methodology rather than a systematic review. This more flexible approach allowed us to reveal how names for concepts are used interchangeably across disciplines. For example, we found some studies using the terms spillovers and unintended consequences interchangeably. We do not aspire to resolve this semantic dispute, but by conceptualising spillovers as a broader overarching set of effects, we offer researchers a framework that should allow them to use a common language.

Along the same lines, we deliberately focused our scoping review on the healthcare literature. This provides a limited overview compared to the universe of research on health implementation. Nevertheless, given our goal of mapping the use of a specific term in a multidisciplinary research area, we believe our choice strikes the balance between comprehensiveness and conciseness.

Within implementation science there is currently much interest in understanding the mechanisms of implementation strategies, the processes through which they produce desired effects and how they can be measured [62]. There is however little focus on how implementation strategies may deliver unintended effects and whether or how these can be mitigated. We highlight the importance of understanding the mechanisms underpinning spillover effects to define whether the spillover is important and relevant for the evaluation of the underlying intervention or policy, echoing similar earlier calls by Angelucci and Maro [18]. Our paper offers an overview of the mechanisms suggested in the literature to date. This can be a useful starting point for researchers and policymakers interested in designing interventions which maximise positive (and minimise negative) impacts in the domain of healthcare or designing evaluations ensuring that (i) all relevant impacts are captured and (ii) appropriate control groups are defined. Furthermore, the extent to which spillover effects can be modelled by analysts remains of interest for the continuous improvement of intervention design. To this end, future research may explore the regularities between mechanisms and theoretical underpinnings of different spillover classes which we have highlighted.

In the review, we also reported evidence that may indicate a substantial bias towards reporting statistically significant spillover effects. This bias limits our interpretation of the results of the review: our discussion is based only on papers which mostly report statistically significant spillover effects. We are unable to detect the instances where potential spillover effects have been examined but not found. A corollary of our INTENTS framework is the suggestion to always evaluate and report the results for all known or expected spillover effects, in addition to the main outcomes addressed by the intervention/policy. This seems a necessary condition to obtain a comprehensive evaluation of intervention impact.

## Conclusions

The complex and interlinked nature of healthcare organisation and delivery means that interventions often have consequences beyond those initially intended. The potential existence of such spillover effects has important consequences for intervention design, evaluation, and decision-making.

The INTENTS framework provides a means for researchers and policymakers to think systematically about the potential spillover effects that interventions in healthcare may cause and the steps they may take when attempting to design and evaluate interventions generating such spillovers. We hope that the application of our framework will enable spillover effects to be planned for a priori in future policymaking and evaluation design and ensure that the full range of costs and benefits of interventions are appropriately identified.

## Supplementary Information

**Additional file 1.** Preferred Reporting Items for Systematic reviews and Meta-Analyses extension for Scoping Reviews (PRISMA-ScR) Checklist.

**Additional file 2.** Search strategy.

**Additional file 3.** Detailed list of papers included in the scoping review.

## Data Availability

Not applicable.

## Abbreviations

EPOC: Effective Practice and Organisation of Care
JEL: Journal of Economic Literature
QOF: Quality and Outcomes Framework
