# Supplementary material for: Framework for identification and measurement of spillover effects in policy implementation: intended non-intended targeted non-targeted spillovers (INTENTS)
Source: Implement Sci Commun. 2022 Mar 14;3:30. doi: 10.1186/s43058-022-00280-8 (PMC8919154; doi:10.1186/s43058-022-00280-8)
Supplement: Supplementary file 2 — Additional file 2. Search strategy. [file 43058_2022_280_MOESM2_ESM.docx]

**Additional file 2 – Search strategy**

Database: Ovid MEDLINE(R) <1946 to October Week 2 2020>, Embase <1974 to 2020 Week 42>

Search Strategy:

--------------------------------------------------------------------------------

1 (((spillover* or spill-over* or "spill over" or "spill overs") and (healthcare$ or health* or care)) not (transmission or zoonotic or animal* or wildlife or virus or bacteri* or parasit* or infecti* or vaccine* or immunis* or immuniz*)).af. (2441)

2 limit 1 to english language (2417)

3 limit 2 to yr="1991 - 2020" (2342)

4 remove duplicates from 3 (1500)
