# Supplementary material for: Framework for identification and measurement of spillover effects in policy implementation: intended non-intended targeted non-targeted spillovers (INTENTS)
Source: Implement Sci Commun. 2022 Mar 14;3:30. doi: 10.1186/s43058-022-00280-8 (PMC8919154; doi:10.1186/s43058-022-00280-8)
Supplement: Supplementary file 3 — Additional file 3. Detailed list of papers included in the scoping review. [file 43058_2022_280_MOESM3_ESM.docx]

**Additional file 3 – Detailed list of papers included in the scoping review**

Type of interventions featured in the articles included in the scoping review, grouped according to the EPOC taxonomy and with citation

| **EPOC Taxonomy** | **N** | **Type of intervention** |
| --- | --- | --- |
| Financial Arrangements  Changes in how funds are collected, insurance schemes, how services are purchased, and the use of targeted financial incentives or disincentives | 57 | Hospital readmission reduction program^1–7^, Results-based financing^8–11^, Provider payment models or fees (DRG, capitation, FFS)^12–23^, Disease-specific financial incentive programs^24–26^, Financial interventions (microcredit, incentives)^27–32^, Insurance coverage expansion^33–53^, Changes in healthcare prices or coverage for patients^54–57^ |
| Implementation Strategies  Interventions designed to bring about changes in healthcare organizations, the behaviour of healthcare professionals or the use of health services by healthcare recipients | 27 | Capability-enhancing interventions (training, health promotion, health communication)^58–79^, Provider/Caregiver training^80–82^, Treatment guidelines^83,84^ |
| Governance Arrangements  Rules or processes that affect the way in which powers are exercised, particularly with regard to authority, accountability, openness, participation, and coherence | 21 | Prescribing recommendations/guidelines or changes to prescription drugs lists^85–93^, Extended access to treatment^94–96^, Gatekeeping/Managed care^97,98^, Public reporting of hospital output/quality information^99,100^, Extended healthcare benefits for specific populations (eg. minorities, veterans)^101–105^ |
| Delivery Arrangements  Changes in how, when and where healthcare is organized and delivered, and who delivers healthcare. | 17 | Closure/opening of hospitals/wards or drug stores^106,107^, E-health technology^108,109^, Changes in care process (group treatment, outreach programs, etc.)^110–112^, Screening programs^113^, Public health measures (insecticide-treated bednets, purified water, living conditions, etc.) ^114–117^, Clinical (or similar) interventions^118–122^ |

**Full references for papers included in the scoping review**

1. Gai Y, Pachamanova D. Impact of the Medicare hospital readmissions reduction program on vulnerable populations. *BMC Health Services Research*. 2019;19(1).

2. Mehtsun WT, Papanicolas I, Zheng J, Orav EJ, Lillemoe KD, Jha AK. National Trends in Readmission Following Inpatient Surgery in the Hospital Readmissions Reduction Program Era. *Annals of Surgery*. 2018;267(4):599-605.

3. Demiralp B, He F, Koenig L. Further Evidence on the System-Wide Effects of the Hospital Readmissions Reduction Program. *Health Services Research*. 2018;53(3):1478-1497. doi:10.1111/1475-6773.12701

4. Ibrahim AM, Nathan H, Thumma JR, Dimick JB. Impact of the Hospital Readmission Reduction Program on Surgical Readmissions Among Medicare Beneficiaries. *Annals of Surgery*. 2017;266(4):617-624.

5. Carey K, Lin M-Y. Readmissions To New York Hospitals Fell For Three Target Conditions From 2008 To 2012, Consistent With Medicare Goals. *Health Affairs*. 2015;34(6):978-985.

6. Kim JW, Mannalithara A, Sehgal M, Mithal A, Singh G, Ladabaum U. A nationwide analysis of readmission rates after colorectal cancer surgery in the US in the Era of the Affordable Care Act. *The American Journal of Surgery*. 2020;220(4):1015-1022. doi:10.1016/j.amjsurg.2020.04.013

7. Lee MS, Hollenbeck BK, Oerline MK, et al. Spillover Effects of the Hospital Readmissions Reduction Program on Radical Cystectomy Readmissions. *Urology Practice*. 2019;6(6):350-356. doi:10.1097/UPJ.0000000000000042

8. Zhang H, Doorslaer E van, Xu L, Zhang Y, Klundert J van de. Can a results-based bottom-up reform improve health system performance? Evidence from the rural health project in China. *Health Economics*. 2019;28(10):1204-1219. doi:10.1002/hec.3935

9. Fichera E, Gray E, Sutton M. How do individuals’ health behaviours respond to an increase in the supply of health care? Evidence from a natural experiment. *Social Science & Medicine*. 2016:170-179.

10. Kristensen SR, Meacock R, Turner AJ, et al. Long-Term Effect of Hospital Pay for Performance on Mortality in England. *New England Journal of Medicine*. 2014;371(6):540-548. doi:10.1056/NEJMoa1400962

11. Sutton M, Elder R, Guthrie B, Watt G. Record rewards: the effects of targeted quality incentives on the recording of risk factors by primary care providers. *Health Economics*. 2010;19(1):1-13.

12. Einav L, Finkelstein A, Ji Y, Mahoney N. Randomized trial shows healthcare payment reform has equal-sized spillover effects on patients not targeted by reform. *Proceedings of the National Academy of Sciences*. 2020;117(32):18939-18947. doi:10.1073/pnas.2004759117

13. Kim SJ, Han K-T, Kim W, Kim SJ, Park E-C. Early Impact on Outpatients of Mandatory Adoption of the Diagnosis-Related Group-Based Reimbursement System in Korea on Use of Outpatient Care: Differences in Medical Utilization and Presurgery Examination. *Health Services Research*. 2018;53(4):2064-2083. doi:10.1111/1475-6773.12749

14. Henke RM, Karaca Z, Gibson TB, Cutler E, White C, Wong HS. Medicare Advantage Penetration and Hospital Costs Before and After the Affordable Care Act. *Medical Care*. 2018;56(4):321-328.

15. Lin E, Cheng XS, Chin K-K, et al. Home Dialysis in the Prospective Payment System Era. *JASN*. 2017;28(10):2993-3004. doi:10.1681/ASN.2017010041

16. Angalakuditi M, Gomes J. Retrospective drug utilization review: impact of pharmacist interventions on physician prescribing. *Clinicoecon Outcomes Res*. 2011;3:105-108. doi:10.2147/CEOR.S21789

17. Rice T, Stearns SC, Pathman DE, DesHarnais S, Brasure M, Tai-Seale M. A tale of two bounties: the impact of competing fees on physician behavior. *Journal of Health Politics, Policy and Law*. 1999;24(6):1307-1330. doi:10.1215/03616878-24-6-1307

18. Zhang M, Booth BM, Smith GRJ. Services utilization before and after the prospective payment system by patients with somatization disorder. *Journal of Behavioral Health Services*. 1998;25(1):76-82.

19. Scheffler RM, Clement DG, Sullivan SD, Hu T, Sung H-Y. The Hospital Response to Medicare’s Prospective Payment System: An Econometric Model of Blue Cross and Blue Shield Plans. *Medical Care*. 1994;32(5):471-485.

20. Muller A. Medicare prospective payment reforms and hospital utilization. Temporary or lasting effects? *Medical Care*. 1993;31(4):296-308.

21. Mitchell JM, Hadley J, Gaskin DJ. Spillover effects of Medicare fee reductions: evidence from ophthalmology. *Journal of Health Care Finance*. 2002;2(3):171-188.

22. Hinde JM, West N, Arbes SJ, Kluckman M, West SL. Did Arkansas’ Medicaid Patient-Centered Medical Home Program Have Spillover Effects on Commercially Insured Enrollees? *INQUIRY*. 2020;57:0046958019900753. doi:10.1177/0046958019900753

23. Cook A, Averett S. Do hospitals respond to changing incentive structures? Evidence from Medicare’s 2007 DRG restructuring. *Journal of Health Economics*. 2020;73:102319. doi:10.1016/j.jhealeco.2020.102319

24. Liu D, Green E, Kasteridis P, et al. Incentive schemes to increase dementia diagnoses in primary care in England: a retrospective cohort study of unintended consequences. *Br J Gen Pract*. 2019;69(680):e154-e163. doi:10.3399/bjgp19X701513

25. Hysong SJ, SoRelle R, Broussard Smitham K, Petersen LA. Reports of unintended consequences of financial incentives to improve management of hypertension. *PloS One*. 2017;12(9):e0184856. doi:10.1371/journal.pone.0184856

26. Muhlestein DB, Wickizer T, Shoben A. The Spillover Effect of a Change in Medicare Reimbursements on Provider Behavior in the Non-Medicare Population for Bariatric Surgery. *World Medical & Health Policy*. 2016;8(1):74-94. doi:10.1002/wmh3.178

27. Dehingia N, Singh A, Raj A, McDougal L. More than credit: Exploring associations between microcredit programs and maternal and reproductive health service utilization in India. *SSM Popul Health*. 2019;9. doi:10.1016/j.ssmph.2019.100467

28. Srinivasan M, Pooler JA. Cost-Related Medication Nonadherence for Older Adults Participating in SNAP, 2013-2015. *American Journal of Public Health*. 2018;108(2):224-230. doi:10.2105/AJPH.2017.304176

29. Undurraga EA, Behrman JR, Leonard WR, Godoy RA. The effects of community income inequality on health: Evidence from a randomized control trial in the Bolivian Amazon. *Social Science & Medicine*. 2016;149:66-75. doi:10.1016/j.socscimed.2015.12.003

30. Handa S, Halpern CT, Pettifor A, Thirumurthy H. The Government of Kenya’s Cash Transfer Program Reduces the Risk of Sexual Debut among Young People Age 15-25. *PLoS One*. 2014;9(1). doi:10.1371/journal.pone.0085473

31. Shei A, Costa F, Reis MG, Ko AI. The impact of Brazil’s Bolsa Família conditional cash transfer program on children’s health care utilization and health outcomes. *BMC Int Health Hum Rights*. 2014;14:10. doi:10.1186/1472-698X-14-10

32. Guerrero N, Molina O, Winkelried D. Conditional cash transfers, spillovers, and informal health care: Evidence from Peru. *Health Economics*. 2020;29(2):111-122. doi:10.1002/hec.3956

33. Kino S, Sato K, Kawachi I. Spillover benefit of improved access to healthcare on reducing worry about housing and meal affordability. *Int J Equity Health*. 2018;17(1):174. doi:10.1186/s12939-018-0877-y

34. Kobayashi LC, Altindag O, Truskinovsky Y, Berkman LF. Effects of the Affordable Care Act Medicaid Expansion on Subjective Well-Being in the US Adult Population, 2010-2016. *Journal of Public Health*. 1236;109(9):1236-1242.

35. Himmelstein G. Effect of the Affordable Care Act’s Medicaid Expansions on Food Security, 2010-2016. *Journal of Public Health*. 1243;109(9):1243-1248.

36. Chen A, Lo Sasso AT, Richards MR. Supply-side effects from public insurance expansions: Evidence from physician labor markets. *Health Economics*. 2018;27(4):690-708.

37. Venkataramani M, Pollack CE, Roberts ET. Spillover Effects of Adult Medicaid Expansions on Children’s Use of Preventive Services. *Pediatrics*. 2017;140(6).

38. Shane DM, Wehby GL. The Impact of the Affordable Care Act’s Dependent Coverage Mandate on Use of Dental Treatments and Preventive Services. *Medical Care*. 2017;55(9):841-847.

39. Hudson JL, Moriya AS. Medicaid Expansion For Adults Had Measurable ‘Welcome Mat’ Effects On Their Children. *Health Affairs*. 2017;36(9):1643-1651. doi:10.1377/hlthaff.2017.0347

40. Li X, Ye J. The spillover effects of health insurance benefit mandates on public insurance coverage: Evidence from veterans. *Journal of Health Economics*. 2017:45-60.

41. Woode ME. Parental health shocks and schooling: The impact of mutual health insurance in Rwanda. *Social Science & Medicine*. 2017;173:35-47. doi:10.1016/j.socscimed.2016.11.023

42. Sommers BD, Chua K-P, Kenney GM, Long SK, McMorrow S. California’s Early Coverage Expansion under the Affordable Care Act: A County-Level Analysis. *Health Services Research*. 2016;51(3):825-845.

43. Shane DM, Ayyagari P. Spillover Effects of the Affordable Care Act? Exploring the Impact on Young Adult Dental Insurance Coverage. *Health Services Research*. 2015;50(4):1109-1124. doi:10.1111/1475-6773.12266

44. Schwartz AL, Sommers BD. Moving For Medicaid? Recent Eligibility Expansions Did Not Induce Migration From Other States. *Health Affairs*. 2014;33(1):88-94. doi:10.1377/hlthaff.2013.0910

45. Vujicic M, Yarbrough C, Nasseh K. The Effect of the Affordable Care Act’s Expanded Coverage Policy on Access to Dental Care. *Medical Care*. 2014;52(8):715–719. doi:10.1097/MLR.0000000000000168

46. Sommers BD, Kenney GM, Epstein AM. New Evidence On The Affordable Care Act: Coverage Impacts Of Early Medicaid Expansions. *Health Affairs*. 2014;33(1):78-87. doi:10.1377/hlthaff.2013.1087

47. Sheu J-T, Lu J-FR. The spillover effect of National Health Insurance on household consumption patterns: evidence from a natural experiment in Taiwan. *Social Science & Medicine*. June 2014:41-49.

48. Joynt KE, Chan D, Orav EJ, Jha AK. Insurance expansion in Massachusetts did not reduce access among previously insured Medicare patients. *Health Affairs*. 2013;32(3):571-578.

49. Atherly A, Coulam RF, Dowd BE, Guy G. The effect of adult HIFA waiver expansions on insurance coverage of children. *Medical Care Research & Review*. 2012;69(4):397-413.

50. White C. A comparison of two approaches to increasing access to care: expanding coverage versus increasing physician fees. *Health Services Research*. June 2012:963-983.

51. Bian J, Lipscomb J, Mello MM. Spillover effects of state mandated benefit laws: the case of outpatient breast cancer surgery. *Inquiry*. 2009;46(4):433-447.

52. Blunt EO, Maclean JC, Popovici I, Marcus SC. Public insurance expansions and mental health care availability. *Health Services Research*. 2020;55(4):615-625. doi:10.1111/1475-6773.13311

53. Shane DM, Wehby GL. Were Patient Protection and Affordable Care Act spillover gains to private dental coverage for dependents widely shared?: An analysis using Medical Expenditure Panel Survey data. *The Journal of the American Dental Association*. 2020;151(3):182-189. doi:10.1016/j.adaj.2019.11.014

54. Walsh B, Nolan A, Brick A, Keegan C. Did the expansion of free GP care impact demand for Emergency Department attendances? A difference-in-differences analysis. *Social Science & Medicine (1982)*. 2019;222:101-111. doi:10.1016/j.socscimed.2018.12.029

55. Kenney G, Marton J, McFeeters J, Costich J. Assessing Potential Enrollment and Budgetary Effects of SCHIP Premiums: Findings from Arizona and Kentucky. *Health Serv Res*. 2007;42(6 Pt 2):2354-2372. doi:10.1111/j.1475-6773.2007.00772.x

56. Lagarde M, Barroy H, Palmer N. Assessing the Effects of Removing User Fees in Zambia and Niger. *J Health Serv Res Policy*. 2012;17(1):30-36. doi:10.1258/jhsrp.2011.010166

57. Wang C, Sweetman A. Delisting eye examinations from public health insurance: Empirical evidence from Canada regarding impacts on patients and providers. *Health Policy*. 2020;124(5):540-548. doi:10.1016/j.healthpol.2020.03.006

58. Dougherty L, Stammer E, Derbile E, et al. A Mixed-Methods Evaluation of a Community-Based Behavior Change Program to Improve Maternal Health Outcomes in the Upper West Region of Ghana. *Journal of Health Communication*. 2018;23(1):80-90.

59. Raza WA, Van de Poel E, Van Ourti T. Impact and spill-over effects of an asset transfer program on child undernutrition: Evidence from a randomized control trial in Bangladesh. *Journal of Health Economics*. 2018;62:105-120. doi:10.1016/j.jhealeco.2018.09.011

60. Cliff BQ, Hirth RA, Mark Fendrick A. Spillover Effects From A Consumer-Based Intervention To Increase High-Value Preventive Care. *Health Affairs*. 2019;38(3):448-455. doi:10.1377/hlthaff.2018.05015

61. Bhadhuri A, Al-Janabi H, Jowett S, Jolly K. Incorporating Household Spillovers in Cost Utility Analysis: A Case Study Using Behavior Change in COPD. *International Journal of Technology Assessment in Health Care*. 2019;35(3):212-220. doi:10.1017/S026646231900028X

62. Hoddinott J, Ahmed I, Ahmed A, Roy S. Behavior change communication activities improve infant and young child nutrition knowledge and practice of neighboring non-participants in a cluster-randomized trial in rural Bangladesh. *PLOS ONE*. 2017;12(6):e0179866. doi:10.1371/journal.pone.0179866

63. Vable AM, Kawachi I, Canning D, Glymour MM, Jimenez MP, Subramanian SV. Are There Spillover Effects from the GI Bill? The Mental Health of Wives of Korean War Veterans. *PLOS ONE*. 2016;11(5):e0154203. doi:10.1371/journal.pone.0154203

64. Coffield E, Nihiser AJ, Sherry B, Economos CD. Shape Up Somerville: change in parent body mass indexes during a child-targeted, community-based environmental change intervention. *American Journal of Public Health*. 2015;105(2):e83-89. doi:10.2105/AJPH.2014.302361

65. Swinburn B, Malakellis M, Moodie M, et al. Large reductions in child overweight and obesity in intervention and comparison communities 3 years after a community project. *Pediatric Obesity*. 2014;9(6):455-462.

66. Tan ASL. Potential spillover educational effects of cancer-related direct-to-consumer advertising on cancer patients’ increased information seeking behaviors: results from a cohort study. *Journal of Cancer Education*. 2014;29(2):258-265.

67. Goebbels AFG, Lakerveld J, Ament AJHA, Bot SDM, Severens JL. Exploring non-health outcomes of health promotion: the perspective of participants in a lifestyle behaviour change intervention. *Health Policy*. 2012;106(2):177-186.

68. de Heer HD, Koehly L, Pederson R, Morera O. Effectiveness and spillover of an after-school health promotion program for hispanic elementary school Children. *American journal of public health*. 2011;101(10):1907-1913. doi:10.2105/AJPH.2011.300177

69. Mata J, Silva MN, Vieira PN, et al. Motivational “spill-over” during weight control: increased self-determination and exercise intrinsic motivation predict eating self-regulation. *Health Psychology*. 2009;28(6):709-716.

70. Gansky SA, Ellison JA, Rudy D, et al. Cluster-Randomized Controlled Trial of An Athletic Trainer-Directed Spit (Smokeless) Tobacco Intervention for Collegiate Baseball Athletes: Results After 1 Year. *J Athl Train*. 2005;40(2):76-87.

71. Sarma EA, Moyer A, Messina CR, et al. Is There a Spillover Effect of Targeted Dietary Change on Untargeted Health Behaviors? Evidence From a Dietary Modification Trial. *Health Educ Behav*. 2019;46(4):569-581. doi:10.1177/1090198119831756

72. Blakstad MM, Bellows AL, Mosha D, et al. Neighbour home gardening predicts dietary diversity among rural Tanzanian women. *Public Health Nutrition*. 2019;22(9):1646-1653. doi:10.1017/S1368980018003798

73. Lowenstein C, Dow WH, White JS. Peer effects in smoking cessation: An instrumental variables analysis of a worksite intervention in Thailand. *SSM - Population Health*. 2020;12:100659. doi:10.1016/j.ssmph.2020.100659

74. Heerman WJ, Samuels LR, Barr L, Burgess LE, Hartmann KE, Barkin SL. The Effect of a General Healthy Lifestyle Intervention Delivered Around Pregnancy on Gestational Weight Gain and Infant Growth. *Maternal and Child Health Journal*. 2020;24(11):1404-1411. doi:10.1007/s10995-020-02998-0

75. Steeves S, Acciai F, Tasevska N, DeWeese RS, Yedidia MJ, Ohri-Vachaspati P. The Special Supplemental Nutrition Program for Women, Infants, and Children Spillover Effect: Do Siblings Reap the Benefits? *Journal of the Academy of Nutrition and Dietetics*. 2020;120(8):1288-1294. doi:10.1016/j.jand.2020.02.013

76. Øberg GK, Girolami GL, Campbell SK, et al. Effects of a Parent-Administered Exercise Program in the Neonatal Intensive Care Unit: Dose Does Matter—A Randomized Controlled Trial. *Phys Ther*. 2020;100(5):860-869. doi:10.1093/ptj/pzaa014

77. Ferreira CM, Goldszmidt R, Andrade EB. The short- and long-term impact of an incentive intervention on healthier eating: a quasi-experiment in primary- and secondary-school cafeterias in Brazil. *Public Health Nutrition*. 2019;22(9):1675-1685. doi:10.1017/S1368980019000223

78. Ruggiero CF, Hohman EE, Birch LL, Paul IM, Savage JS. The Intervention Nurses Start Infants Growing on Healthy Trajectories (INSIGHT) responsive parenting intervention for firstborns impacts feeding of secondborns. *Am J Clin Nutr*. 2020;111(1):21-27. doi:10.1093/ajcn/nqz277

79. Dillon A, Bliznashka L, Olney D. Experimental evidence on post-program effects and spillovers from an agriculture-nutrition program. *Economics & Human Biology*. 2020;36:100820. doi:10.1016/j.ehb.2019.100820

80. Harrop C, Gulsrud A, Shih W, Hovsepyan L, Kasari C. The impact of caregiver-mediated JASPER on child restricted and repetitive behaviors and caregiver responses. *Autism Research*. 2017;10(5):983-992. doi:10.1002/aur.1732

81. Simmons RK, Bruun NH, Witte DR, et al. Does training of general practitioners for intensive treatment of people with screen-detected diabetes have a spillover effect on mortality and cardiovascular morbidity in “at risk” individuals with normoglycaemia? Results from the ADDITION-Denmark cluster-randomised controlled trial. *Diabetologia*. 1016;60(6):1016-1021.

82. Charles M, Skriver MV, Griffin SJ, et al. Does Training and Support of General Practitioners in Intensive Treatment of People with Screen-Detected Diabetes Improve Medication, Morbidity and Mortality in People with Clinically-Diagnosed Diabetes? Investigation of a Spill-Over Effect in a Cluster RCT. *PLOS ONE*. 2017;12(2):e0170697. doi:10.1371/journal.pone.0170697

83. Valuck RJ, Libby AM, Orton HD, Morrato EH, Allen R, Baldessarini RJ. Spillover Effects on Treatment of Adult Depression in Primary Care After FDA Advisory on Risk of Pediatric Suicidality With SSRIs. *AJP*. 2007;164(8):1198-1205. doi:10.1176/appi.ajp.2007.07010007

84. Lyons BA, Merola V, Reifler J. Shifting medical guidelines: Compliance and spillover effects for revised antibiotic recommendations. *Social Science & Medicine*. 2020;255:112943. doi:10.1016/j.socscimed.2020.112943

85. Howard R, Alameddine M, Klueh M, et al. Spillover Effect of Evidence-Based Postoperative Opioid Prescribing. *Journal of the American College of Surgeons*. 2018;227(3):374-381. doi:10.1016/j.jamcollsurg.2018.06.007

86. Grecu AM, Dave DM, Saffer H. Mandatory Access Prescription Drug Monitoring Programs and Prescription Drug Abuse. *Journal of Policy Analysis and Management*. 2019;38(1):181-209. doi:10.1002/pam.22098

87. Han E, Kim TH, Jeung MJ, Lee E-K. Analyses of direct and indirect impacts of a positive list system on pharmaceutical R&D investments. *Clinical Therapeutics*. 2013;35(7):941-949. doi:10.1016/j.clinthera.2013.05.002

88. Lakdawalla D, Sood N, Gu Q. Pharmaceutical advertising and Medicare Part D. *Journal of Health Economics*. 2013;32(6):1356-1367. doi:10.1016/j.jhealeco.2013.01.001

89. Lu CY, Srasuebkul P, Drew AK, Ward RL, Pearson S-A. Positive spillover effects of prescribing requirements: increased cardiac testing in patients treated with trastuzumab for HER2+ metastatic breast cancer. *Internal Medicine Journal*. 2012;42(11):1229-1235. doi:10.1111/j.1445-5994.2011.02604.x

90. Virabhak S, Shinogle JA. Physicians’ prescribing responses to a restricted formulary: the impact of Medicaid preferred drug lists in Illinois and Louisiana. *The American Journal of Managed Care*. 2005;11 Spec No:SP14-20.

91. Wang YR, Pauly MV, Lin YA. Impact of Maine’s Medicaid drug formulary change on non-Medicaid markets: spillover effects of a restrictive drug formulary. *The American Journal of Managed Care*. 2003;9(10):686-696.

92. Wang YR, Pauly MV. Spillover effects of restrictive drug formularies: a case study of PacifiCare in California. *The American Journal of Managed Care*. 2005;11(1):24-26.

93. McGee LM, Kolli A, Harbaugh CM, et al. Spillover Effect of Opioid Reduction Interventions From Adult to Pediatric Surgery. *Journal of Surgical Research*. 2020;249:18-24. doi:10.1016/j.jss.2019.11.021

94. Almeida ATC de, Sá EB de, Vieira FS, et al. Impacts of a Brazilian pharmaceutical program on the health of chronic patients. *Revista de Saúde Pública*. 2019;53. doi:10.11606/s1518-8787.2019053000733

95. Fernandez J, Lang M. Suicide and Organ Donors: Spillover Effects of Mental Health Insurance Mandates. *Health Economics*. 2015;24(4):491-497. doi:10.1002/hec.3037

96. Zuvekas SH, Rupp AE, Norquist GS. Spillover effects of benefit expansions and carve-outs on psychotropic medication use and costs. *Inquiry*. 2005;42(1):86-97.

97. Conover CJ, Rankin PJ, Sloan FA. Effects of Tennessee Medicaid Managed Care on Obstetrical Care and Birth Outcomes. *Journal of Health Politics, Policy and Law*. 2001;26(6):1291-1324.

98. Dumontet M, Buchmueller T, Dourgnon P, Jusot F, Wittwer J. Gatekeeping and the utilization of physician services in France: Evidence on the Médecin traitant reform. *Health Policy*. 2017;121(6):675-682. doi:10.1016/j.healthpol.2017.04.006

99. Nathan Ashwin S., Shah Rohan M., Khatana Sameed A., et al. Effect of Public Reporting on the Utilization of Coronary Angiography After Out-of-Hospital Cardiac Arrest. *Circulation: Cardiovascular Interventions*. 2019;12(4):e007564. doi:10.1161/CIRCINTERVENTIONS.118.007564

100. Li X. Quality information disclosure and health insurance demand: evidence from VA hospital report cards. *Int J Health Econ Manag*. 2020;20(2):177-199. doi:10.1007/s10754-019-09276-9

101. Wong ES, Maciejewski ML, Hebert PL, Fortney JC, Liu C-F. Spillover Effects of Massachusetts Health Reform on Mental Health Use by VA and Medicare Dual Enrollees. *Adm Policy Ment Health*. 2019;46(2):145-153. doi:10.1007/s10488-018-0900-z

102. Allen CD. Who loses public health insurance when states pass restrictive omnibus immigration-related laws? The moderating role of county Latino density. *Health & Place*. 2018:20-28.

103. Fu R, Noguchi H, Kawamura A, Takahashi H, Tamiya N. Spillover effect of Japanese long-term care insurance as an employment promotion policy for family caregivers. *Journal of Health Economics*. 2017:103-112.

104. Boyle MA, Lahey JN. Spousal labor market effects from government health insurance: Evidence from a veterans affairs expansion. *Journal of Health Economics*. 2016;45:63-76. doi:10.1016/j.jhealeco.2015.11.005

105. Morrissey JP, Domino ME, Cuddeback GS. Expedited Medicaid Enrollment, Mental Health Service Use, and Criminal Recidivism Among Released Prisoners With Severe Mental Illness. *Psychiatric Services*. 2016;67(8):842-849.

106. Hsia RY, Shen Y-C. Emergency Department Closures And Openings: Spillover Effects On Patient Outcomes In Bystander Hospitals. *Health Affairs*. 2019;38(9):1496-1504. doi:10.1377/hlthaff.2019.00125

107. Conyers G, Ayres I. A lottery test of the effect of dispensaries on emergency room visits in Arizona. *Health Economics*. 2020;29(8):854-864. doi:10.1002/hec.4013

108. Guo S, Du W, Chen S, Guo X, Ju X. Exploring the Impact of the Rational Antibiotic Use System on Hospital Performance: The Direct Effect and the Spillover Effect. *International Journal of Environmental Research and Public Health*. 2019;16(18). doi:10.3390/ijerph16183463

109. Long T, Bongiovanni T, Dashevsky M, et al. Impact of laboratory cost display on resident attitudes and knowledge about costs. *Postgraduate Medical Journal*. 2016;92(1092):592-596. doi:10.1136/postgradmedj-2015-133851

110. Buchtemann D, Kastner D, Warnke I, et al. Hospital utilization outcome of an assertive outreach model for schizophrenic patients - results of a quasi-experimental study. *Psychiatry Research*. July 2016:249-255.

111. Weigl M, Hornung S, Glaser J, Angerer P. Reduction of Hospital Physicians’ Workflow Interruptions: A Controlled Unit-Based Intervention Study. *Journal of Healthcare Engineering*. 2012;3(4):605-620. doi:10.1260/2040-2295.3.4.605

112. Stokes J, Kristensen SR, Checkland K, Bower P. Effectiveness of multidisciplinary team case management: difference-in-differences analysis. *BMJ Open*. 2016;6(4):e010468. doi:10.1136/bmjopen-2015-010468

113. Miettinen J, Malila N, Hakama M, Pitkaniemi J. Spillover improved survival in non-invited patients of the colorectal cancer screening programme. *Journal of Medical Screening*. 2018;25(3):134-140.

114. Jarvis CI, Multerer L, Lewis D, et al. Spatial Effects of Permethrin-Impregnated Bed Nets on Child Mortality: 26 Years on, a Spatial Reanalysis of a Cluster Randomized Trial. *The American Journal of Tropical Medicine and Hygiene*. 2019;101(6):1434-1441. doi:10.4269/ajtmh.19-0111

115. Kim DA, Hwong AR, Stafford D, et al. Social network targeting to maximise population behaviour change: a cluster randomised controlled trial. *The Lancet*. 2015;386(9989):145-153. doi:10.1016/S0140-6736(15)60095-2

116. Kearns A, Ghosh S, Mason P, Egan M. Urban regeneration and mental health: Investigating the effects of an area-based intervention using a modified intention to treat analysis with alternative outcome measures. *Health & Place*. 2020;61:102262. doi:10.1016/j.healthplace.2019.102262

117. De Shay R, Comeau DL, Sclar GD, Routray P, Caruso BA. Community Perceptions of a Multilevel Sanitation Behavior Change Intervention in Rural Odisha, India. *International Journal of Environmental Research and Public Health*. 2020;17(12):4472. doi:10.3390/ijerph17124472

118. Halliday TM, Davy BM, Clark AG, et al. Dietary intake modification in response to a participation in a resistance training program for sedentary older adults with prediabetes: findings from the Resist Diabetes study. *Eating Behaviors*. 2014;15(3):379-382.

119. Quinto Romani A. Estimating the peer effect on youth overweight and inactivity using an intervention study. *Journal of School Health*. 2014;84(10):617-624.

120. James-Burdumy S, Goesling B, Deke J, Einspruch E. The effectiveness of mandatory-random student drug testing: a cluster randomized trial. *Journal of Adolescent Health*. 2012;50(2):172-178.

121. Kertesz SG, Madan A, Wallace D, Schumacher JE, Milby JB. Substance abuse treatment and psychiatric comorbidity: do benefits spill over? Analysis of data from a prospective trial among cocaine-dependent homeless persons. *Substance Abuse Treatment, Prevention, and Policy*. 2006;1:27. doi:10.1186/1747-597X-1-27

122. Holliday A, Burgin A, Fernandez EV, Fenton SAM, Thielecke F, Blannin AK. Points-based physical activity: a novel approach to facilitate changes in body composition in inactive women with overweight and obesity. *BMC Public Health*. 2018;18(1).
